# 3D comparative analysis of designed versus achieved maxillary teeth movements and influencing factors following first premolar extraction with invisalign: a new digital root model

**DOI:** 10.1186/s40510-025-00569-z

**Published:** 2025-07-01

**Authors:** Waseem S. Al-Gumaei, Reem Al-Attab, Hu Long, Wenli Lai, Fan Jian

**Affiliations:** 1 Department of Orthodontics, State Key Laboratory of Oral Diseases & National Center for Stomatology & National Clinical Research Center for Oral Diseases, West China Hospital of Stomatology, Sichuan University, Chengdu, 610041 China; 2 Department of Orthodontics and Maxillofacial Orthopaedics, College of Dentistry, Ibb University, Ibb, Yemen; 3 State Key Laboratory of Oral Diseases, Department of Cosmetic and Plastic Surgery, Oral and Maxillofacial Surgery, National Clinical Research Center for Oral Diseases, West China Hospital of Stomatology, Sichuan University, Chengdu, 610041 China

**Keywords:** Designed tooth movement, Achieved tooth movement, First premolar extraction, Influencing factors, New digital root model

## Abstract

**Objectives:**

This study aimed to compare the designed and achieved maxillary teeth movements in patients undergoing first premolar extraction after the initial series of Invisalign^®^ treatment using a 3D whole-tooth (crown with root) model and research the related influencing factors.

**Materials and methods:**

Thirty-three consecutive adult patients (Class I with crowding or bimaxillary protrusion) from a single clinical division who completed the first series of aligners after first premolar extractions were included in this retrospective study. The pretreatment, designed, and post-first series treatment teeth (crowns, roots, and bone) models were exported from ClinCheck^®^ software (Align Technology). The superimposition of the models and 3D tooth movement measurements were constructed using Geomagic Studio Software 2014 (Raindrop Geomagic Inc., USA). Descriptive and analytical statistics were performed, and a *P*-value < 0.05 was considered statistically significant.

**Results:**

Significant discrepancies were observed between the designed and achieved maxillary tooth movements, particularly in mesiodistal (except U1) and buccolingual (except U6) angular tooth movements (*P* < 0.05). In contrast, non-significant discrepancies were observed in linear buccolingual movements (*P* > 0.05). Significant discrepancies in mesiodistal (all teeth) and vertical (U1, U5, U6) tooth movements were more pronounced (*P* < 0.05). Key influencing factors included the usage of TADs, aligner generation (G6), attachment design, overbite, and gender (*P* < 0.05).

**Conclusions:**

This study highlights significant deviations between the designed and achieved maxillary teeth (crowns with roots) movements following the first premolar extractions in the initial series of Invisalign^®^treatment. Key findings demonstrate significant mesiodistal (except U1) and buccolingual (except U6) angular movement deviations. While linear buccolingual movements were well-controlled, mesiodistal and vertical (U1, U5, U6) discrepancies were more pronounced, influenced by factors such as TADs usage, aligner generation (G6), attachment design, overbite, and gender. These findings may provide further evidence for virtual design during clear aligner treatment.

**Supplementary Information:**

The online version contains supplementary material available at 10.1186/s40510-025-00569-z.

## Introduction

Clear aligners (CA) are becoming increasingly popular in orthodontic treatment as an alternative to fixed appliances. They offer better aesthetics, comfort, and oral health benefits [1, 2]. CA has recently evolved from treating simple non-extraction malocclusion cases to addressing more complex cases, including those requiring tooth extraction. However, the increased complexity, tooth movement, and treatment duration may pose additional challenges in achieving favourable outcomes regarding the three-dimensional final tooth positions [3].

Understanding the CA efficacy or predictability of tooth movement is essential in treatment goal determination and treatment time and costs assessment. Historically, 3D digital planning of final tooth positions could be designed only based on the crown through ClinCheck^®^ software [4]. To date, small number of studies concerning the efficacy of tooth crown movement in premolar extraction treatment with clear aligners have been conducted [5–9]. For example, Dai et al. considered the comparison between achieved and predicted crown movement in adults after four first premolar extraction treatments at upper and lower central incisors, canines, second premolars, and first molars [6, 10]. In another study, they also considered maxillary central incisors and first molars’ crowns [5]. Recently, they conducted a new study to assess the differences between achieved and predicted root movement in all upper and lower teeth [6, 10]. Feng et al. assessed the difference between the designed and achieved mesiodistal angulation of maxillary canines and posterior teeth [7]. Ren et al. assessed the difference between the designed and achieved three-dimensional crown movements of maxillary incisors, canines, and first molars’ crowns [8]. Thilagalavanian et al. found that the achieved root angulation in teeth adjacent to premolar extraction sites in the maxilla after treatment with an initial series of Invisalign^®^ aligners differed significantly from the predicted [9]. However, these studies have shown that the tooth movements (TM) cannot be fully accomplished as designed. Their assessments relied on the old crown model setup and were less focused on influencing factors. Therefore, the efficacy or predictability of TM based on the whole tooth (crown with root) remains somewhat unclear.

In recent advancements, Invisalign has employed deep learning techniques to combine data from intra-oral scans (crown information) and cone-beam computed tomography (CBCT) scans (root and alveolar bone information). This integration allows for a new comprehensive crown, roots, and alveolar bone model (CRAM), which is used to design precise tooth movements for orthodontic treatment. However, no study has investigated the predictability of such updates in the context of orthodontic treatment planning and the design of individualized interventions. Moreover, the predictably of CA actual tooth movements is influenced by numerous factors, including age, gender, crowding, overjet, overbite, generation (G6), mini-implant, and attachment influence [8, 9], Thus, understanding the influencing factors, degree, and mechanisms of the deviation between the setup design and the achieved TM is essential for orthodontists to design a better CA plan in future.

Therefore, this retrospective study aimed to compare designed and achieved 3D whole tooth (crown with root) movements and their influencing factors for all maxillary teeth in first premolar extraction patients treated with Invisalign^®^ aligners, providing more evidence for virtual design during clear aligner treatment.

## Methods and materials

### Sample selection

A total of 33 orthodontic patients with 396 teeth were retrospectively selected from the Department of Orthodontics at West China Hospital of Stomatology, Sichuan University, with the selection process taking place between October 2022 and September 2024. The sample size was determined utilizing G*power 3.0.10 software with an alpha value of 0.05 and a power of 95% based on the study conducted by Ren et al., the designed canine retraction was 6.5 ± 1.6 mm while the achieved was 5.2 ± 2.2 mm. Power analysis showed a minimum sample size of 27 [8]. The clear aligners (Invisalign^®^, Align Technology, San Jose, Calif) employed G6R (Reinforcement G6, 2020), optimized, vertical, or horizontal attachments. The treatment plan was staged with premolar extractions, molar anchorage preparation involving distal tipping, sequential canine retraction, and en masse retraction of the incisors. Selective incorporation of intra- or inter-arch elastics and posterior buccal temporary anchorage devices (TADs) was applied according to the clinical requirements. All the participants were instructed to wear aligners for at least 22 h/day and to change to the next pair of aligners every 10 days.

The inclusion criteria required: (1) patients aged 18 to 35 years, (2) first premolar extraction (class I with crowding or bimaxillary protrusion), (3) initial CA series with completed extraction space closure (4) no missing teeth, except for third molars, (5) no prior orthodontic or prosthetic treatment, and (6) good patient compliance. The exclusion criteria eliminated patients with: (1) skeletal discrepancies, (2) progressive periodontal disease, (3) endodontic treatments or crown restorations, (4) generalized caries, (5) pathobiological lesions, (6) mesiodens, (7) receiving auxiliary orthodontic appliances to facilitate tooth movement, such as segmental archwire, (8) systematic diseases, or (9) smoking habits.

Ethical approval for this study was granted under approval number “WCHSIRB-CT-2024-320.” Informed consent was obtained from all participants and their parents or legal guardians, when applicable. All methods adhered to the principles outlined in the Helsinki Declaration.

### 3D measurements

**Fig. 1:**
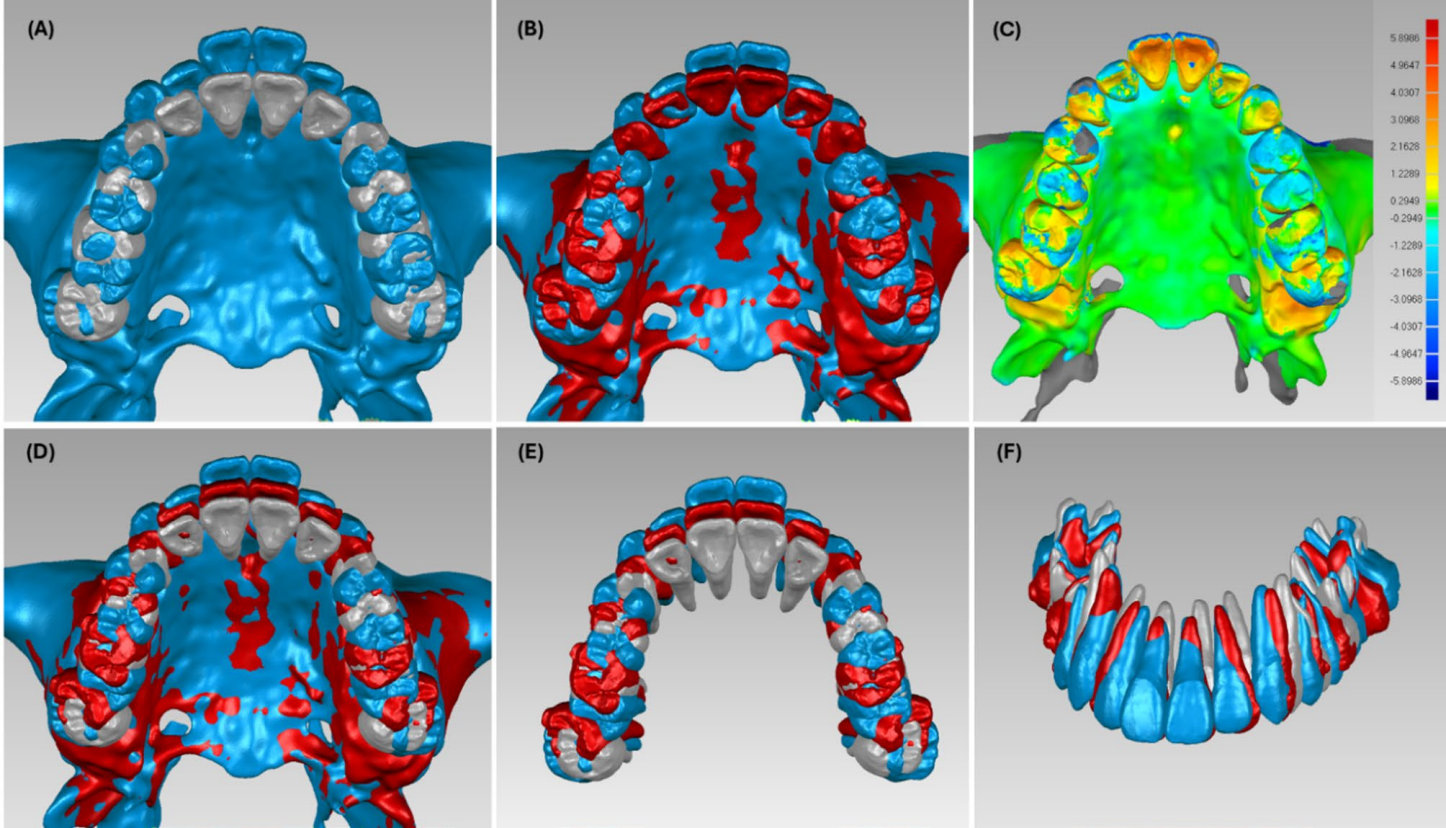
Superimposition of pre- and post-models and establishment of maxillary teeth models. (**A**) Pretreatment (blue) and designed (gray) from ClinCheck; (**B**) Best-fit superimposition of pre-treatment (blue) and achieved (red) models based on maxillary bone; (**C**) Color mapping of Best-fit superimposition for pre-treatment and achieved models; (**D**) Final superimposition of all models; (**E**, **F**) Isolated teeth models

After collecting demographic and clinical data regarding age, gender, attachment, crowding, overjet, overbite, and orthodontic TADs, the pretreatment, designed, and post-initial series treatment achieved models were exported from ClinCheck^®^ software. These models included crowns (obtained from iTero intraoral scanning, Align Technology, San José, CA, USA), roots, and maxillary bone (segmented from CBCT). All models were imported into Geomagic Studio Software 2014 (Raindrop Geomagic Inc., USA). The pretreatment and designed models were superimposed by ClinCheck^®^ software and maintained by Geomagic Studio Software 2014. The pre-treatment and achieved models were superimposed based on the maxillary bone with best-fit features and verified with colour mappings to ensure the accuracy of superimposition. The 3D whole tooth (crowns with roots) models were then extracted by removing the maxillary bone (Fig. 1).

**Fig. 2:**
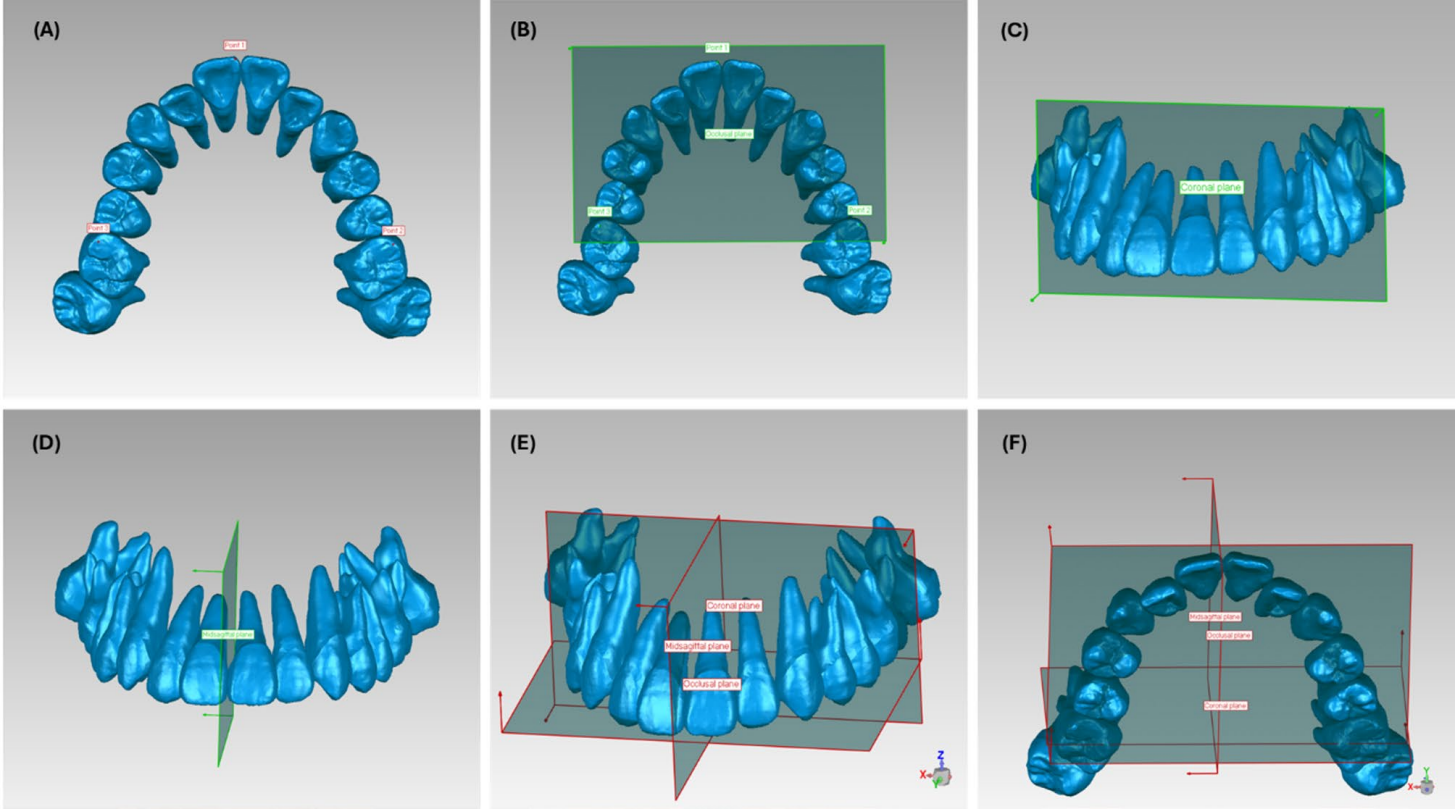
Establishment of the world coordinate system of maxillary teeth. (**A**) The points used to construct the occlusal plane (transverse plane); (**B**) Establishment of the occlusal (transverse) plane; (**C**) Establishment of the coronal plane; (**D**) Establishment of the midsagittal plane; (**E**, **F**) The world coordinate system for maxillary teeth

A world coordinate system was constructed on the pre-treatment model, then transferred to the designed and achieved models to ensure comparability. The coordinate system reference planes were established based on the pre-treatment models. Specifically, the occlusal or transverse plane(X-axis) was defined as a best-fit plane between mesiobuccal cusps of bilateral first molars and the mesioincisal point of the central incisor. The coronal plane (Z-axis) went through a line that passing through the mesiobuccal cusps of the bilateral first molars and was perpendicular to the occlusal plane. Furthermore, the midsagittal plane(Y-axis) was perpendicular to the transverse and coronal planes and passed through the mesioincisal point of the central incisor (Fig. 2) [5, 6, 8].

**Fig. 3:**
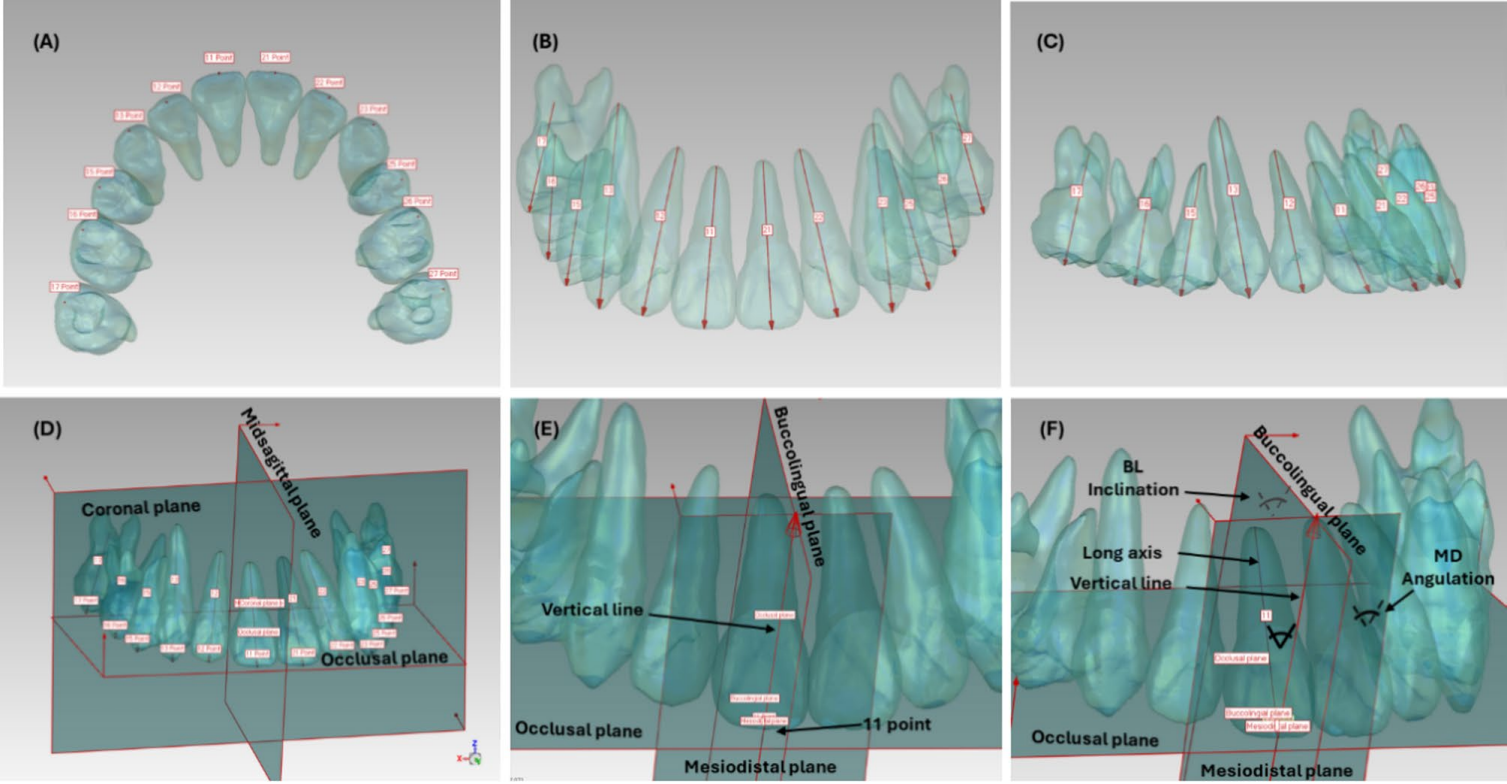
Steps of the 3D measurements of maxillary teeth. (**A**) The points used for measuring linear tooth movements and constructing reference lines/planes; (**B**, **C**) The 3D teeth’s long axes. (**D**) The transported world coordinate system of maxillary teeth on the designed/achieved model; (**E**) Example of the specific-tooth coordinate system; (**F**) Example of the measurement of mesiodistal angulation and buccolingual inclination

A specific tooth coordination system was developed to assess mesiodistal angulation and buccolingual inclination, with tooth reference points and planes established based on each designed and achieved model. The reference points included the midpoint of the incisal edges for the central and lateral incisors, the canine cusp, the buccal cusp of the second premolar, and the mesiobuccal cusp of the first and second molars. The mesiodistal plane passes through the reference point, is perpendicular to the occlusal plane, and is parallel to the coronal plane in anterior teeth or the midsagittal plane in posterior teeth. The buccolingual plane passes through the reference point, is perpendicular to the occlusal plane, and is parallel to the midsagittal plane in anterior teeth or the coronal plane in posterior teeth. The vertical line is defined as the intersection of the mesiodistal and buccolingual planes, passing through the reference point and being perpendicular to the occlusal plane (Fig. 3) [6].

The tooth long axis is defined as the line passing through the midpoint of the incisal edges and the root apex for the central and lateral incisors, through the cusp and root apex of the canine, through the buccal cusp and root apex of the second premolar, and through the mesiobuccal cusps and their root apexes for the first and second molars (Fig. 3) [7].

The angular measurements were performed according to the specific tooth coordination system. For example, the mesiodistal angulation of anterior teeth is measured as the angle between the tooth’s long axis and vertical line projected to the mesiodistal plane, while the buccolingual inclination is measured as the angle between the tooth’s long axis and vertical line projected to the buccolingual plane [6, 8]. The linear measurements were performed according to the reference points (the midpoint of the incisal edges for the central and lateral incisors, the canine cusp, the buccal cusp of the second premolar, and the mesiobuccal cusp of the first and second molars). The reference points were digitized on both designed and achieved models as 3D landmarks on the X, Y, and Z axes (Fig. 3) [7, 8]. After one month, the intra-rater reliability was assessed for all 3D measurements in 10% of the sample.

### Statistical analysis

Data were analysed using SPSS Statistics 26 (Statistical Package for the Social Sciences, SPSS Inc., Chicago, IL, USA) and checked for normal distribution using the Shapiro–Wilk test. A paired t-test was used to compare the differences between designed and achieved tooth movements. A multivariate linear mixed model regression analysis was performed to examine the influence of variables (age, gender, overbite, overjet, TADs, crowding, attachment, and power ridge) on the differences between designed and achieved tooth movements. The intra-class coefficient (ICC) test was used to analyse the inter-rater reliability of the whole 3D measurements. *P*-values less than 0.05 were considered statistically significant.

## Results

A total of 33 patients with 396 teeth were included in this study. The demographic and baseline data of the patients are displayed in Table 1. Only adults participated in this study: 11 males (26.38 ± 6.45 years), 22 females (23.77 ± 5.76 years). As shown in Table 2, the tooth movement measurement’s reliability was “excellent agreement”: ICC above 95% with *P* < 0.05. Table 3 presents the differences between the designed and achieved movements for upper teeth (U1, U2, U3, U5, U6, and U7) across both angular (MD° and BL°) and linear (MD-mm, BL-mm, and IE-mm) measurements. These differences were observed with varying levels of statistical significance.

### Upper teeth angular movements

The results for the upper teeth’s angular movements reveal significant discrepancies between the designed and achieved outcomes for most teeth. For mesiodistal tipping (MD°), significant differences were observed in teeth U2, U3, U5, U6, and U7 (*P* < 0.05 to *P* < 0.001). The achieved movements were greater than the designed movements, U2 and U3 with more distal tipping, while U5, U6, and U7 had more mesial tipping. This suggests that the actual mesiodistal tipping during treatment exceeded the planned values. The data from U1 showed no significant difference (*P* = 0.873), suggesting that the achieved movement closely matched the designed one for this tooth.

For buccolingual inclination (BL⁰), significant differences were found in all upper teeth (*P* < 0.05), except for U6. The achieved movements were less than the designed movements in most upper teeth, with more buccal tooth inclination (lingual root torque). However, the achieved movements were greater than the designed movements in U7 with buccal tooth inclination (lingual root torque). This indicates that the buccolingual inclination achieved during treatment was consistently less than what was planned. These findings highlight challenges in controlling angular movements, particularly in achieving the desired buccolingual inclination.

### Upper teeth linear movements

In terms of mesiodistal linear movements (MD-mm), significant differences were observed in teeth U1, U2, U5, U6, and U7 (*P* < 0.05 to *P* < 0.01), with achieved movements being greater than the designed movements in most teeth: U1 and U2 with more distal movement, and U5, U6, and U7 with more mesial movement. The achieved MD-mm movements of U3 were slightly greater than designed, but without a statistically significant difference (*P* > 0.05). This suggests that the teeth moved more mesiodistally than planned. For buccolingual linear movements (BL-mm), no significant differences were found in any of the upper teeth (*P* > 0.05), indicating that the achieved buccolingual linear movements were consistent with the designed values. However, for intrusion/extrusion (IE-mm), significant differences were observed in teeth U1, U5, and U6 (*P* < 0.05 to *P* < 0.01). The achieved IE-mm movements were greater than those designed in U1 with more extrusion, while the achieved movements were less than those designed in U5 (with more extrusion) and U6 (with intrusion). This suggests that vertical movements were not as pronounced as planned, particularly in these teeth. The other teeth had no significant differences and well-controlled vertical movements. Overall, linear movements in the buccolingual dimension were well-controlled; however, mesiodistal and vertical movements showed greater deviations from the planned outcomes.

### Upper teeth movements influencing factors

The multivariate linear mixed model regression analysis results (Supplementary Tables 4–9) showed the impact of various factors (age, gender, overbite (OB), overjet (OJ), temporary anchorage devices (TADs), crowding, G6, attachment type, and power ridge) on the discrepancies between designed and achieved upper teeth movements. The findings for each tooth are summarized as below:

### Upper central incisor (U1)

The multivariate linear mixed model regression analysis for the upper central incisor revealed several significant influencing factors on its movement (Supplementary Table 4). Overjet (OJ) was found to have a statistically significant positive effect on the mesiodistal angular (MD°) movement discrepancies (β = 0.34, *P* = 0.015), while overbite (OB) negatively impacted the MD° discrepancies (β = −0.45, *P* = 0.032), positively impacted buccolingual angular (BL°) movement discrepancies (β = 1.19, *P* = 0.001), and positively affected intrusion-extrusion linear (IE-mm) movement discrepancies (β = 0.31, *P* = 0.022). Crowding was positively associated with a significant increase in buccolingual linear (BL-mm) movement discrepancies (β = 0.08, *P* = 0.025). The absence of a temporary anchorage devices (TADs) was significantly correlated with reductions in mesiodistal linear (MD-mm) movement discrepancies (β = −1.79, *P* = 0.005) and increases in the buccolingual linear (BL-mm) movement discrepancies compared to the presence of TADs (β = 0.53, *P* = 0.047). Male gender was linked to greater buccolingual linear (BL-mm) movement discrepancies than females (β = 0.48, *P* = 0.016). Furthermore, the absence of Generation 6 (G6) was significantly associated with a decrease in both the BL° (β = −5.63, *P* = 0.006) and BL-mm discrepancies (β = −1.34, *P* < 0.001). The absence of attachments was linked to significant decreases in the BL° discrepancies (β = −3.41, *P* = 0.003) and BL-mm movement discrepancies, compared to the power ridge (PR) (β = −0.41, *P* = 0.030). Age did not exhibit a significant association with any movement parameters (*P* > 0.05 for all). These findings underscore OB, TADs status, G6, and attachments presence as key determinants in achieving predictable orthodontic outcomes.

### Upper lateral incisor (U2)

The multivariate linear mixed model regression analysis (Supplementary Table 5) revealed several significant associations across different parameters for the upper lateral incisor. OB significantly influenced both the mesiodistal angular (MD⁰) (β = −1.17, *P* = 0.011) and the buccolingual angular (BL⁰) discrepancies (β = 1.25, *P* = 0.007), with a negative effect on the MD⁰ angle and a positive impact on the BL⁰ discrepancies. The absence of TADs was significantly associated with an increase in the BLº discrepancies (β = 4.81, *P* = 0.029) and a reduction in the mesiodistal linear (MD-mm) movement discrepancies (β = −2.40, *P* < 0.001). Male gender was linked to a significant increase in buccolingual linear (BL-mm) movement discrepancies compared to females (β = 0.73, *P* = 0.014). The absence of G6 was associated with a significant decrease in BL-mm discrepancies (β = −1.52, *P* = 0.002). Additionally, the lack of attachment significantly increased MD-mm discrepancies (β = 1.04, *P* = 0.049). At the same time, the presence of the power ridge (PR) also showed an effect on MD-mm discrepancies compared to One Optimized Attachment, a significant positive (O1) (β = 1.17, *P* = 0.024). Age, OJ, and crowding did not demonstrate significant associations with any of the movement parameters (*P* > 0.05 for all). These findings highlight OB, TADs status, gender, G6, and attachment type as crucial factors for optimized treatment outcomes.

### Upper canine (U3)

In the case of the upper canine, the multivariate linear mixed model regression analysis identified several significant associations (Supplementary Table 6). OB significantly influenced the mesiodistal angular (MD⁰) discrepancies (β = −1.08, *P =* 0.003), with a negative effect. Age showed a significant positive association with intrusion-extrusion linear (IE-mm) movement discrepancies (β = 0.04, *P* = 0.033). The absence of TADs was significantly associated with an increase in IE-mm discrepancies compared to the presence of TADs (β = 1.14, *P* = 0.003). Male gender was linked to a significant decrease in IE-mm discrepancies compared to females (β = −0.73, *P* = 0.005). The absence of G6 was associated with a significant increase in the MD⁰ discrepancies (β = 4.70, *P* = 0.021). Additionally, the presence of one optimized attachment (O1) significantly increased IE-mm discrepancies compared to the 3 mm vertical attachment (V3) (β = 1.27, *P* = 0.003). OJ, crowding, and the presence of two optimized attachments (O2) did not demonstrate significant associations with any of the movement parameters (*P* > 0.05 for all). These findings highlight OB, age, TADs status, gender, G6, and attachment type as critical determinants for optimizing orthodontic treatment outcomes for the upper canine.

### Upper second premolar (U5)

The analysis for the upper second premolar (Supplementary Table 7) revealed that crowding significantly influenced the mesiodistal angular (MD⁰) discrepancies (β = 1.08, *P* = 0.003), with a positive effect. OJ showed a significant negative association with the mesiodistal linear (MD-mm) movement discrepancies (β = −0.23, *P* = 0.011) and a marginal negative association with intrusion-extrusion linear (IE-mm) movement discrepancies (β = −0.10, *P* = 0.048). OB was significantly associated with increased MD-mm discrepancies (β = 0.49, *P* = 0.002). The absence of TADs was significantly associated with reducing MD-mm discrepancies (β = −1.94, *P* = 0.005). The absence of G6 was associated with a significant decrease in the MD⁰ discrepancies (β = −8.50, *P* = 0.004). The presence of one optimized attachment (O1) significantly increased MD-mm discrepancies (β = 3.02, *P* = 0.002). Additionally, the presence of a 3 mm horizontal attachment (H3) showed a marginal positive effect on the buccolingual linear (BL-mm) movement discrepancies, compared to the 3 mm vertical attachment (V3) (β = 0.77, *P* = 0.047). Age, gender, and the presence of 3 mm vertical attachment (V3) did not demonstrate significant associations with any of the movement parameters (*P* > 0.05 for all). These findings emphasize the importance of considering crowding, OJ, OB, TADs status, G6, and attachment type to optimize orthodontic treatment outcomes for the upper second premolar.

### Upper first molar (U6)

For the upper first molar, the multivariate linear mixed model regression analysis (Supplementary Table 8) indicated that age showed a significant positive association with intrusion-extrusion linear (IE mm) movement discrepancies (β = 0.05, *P* = 0.020). OB was significantly associated with an increase in mesiodistal linear (MD-mm) movement discrepancies (β = 0.64, *P* = 0.017). The absence of TADs was significantly associated with an increase in the mesiodistal angular (MD°) discrepancies (β = 6.30, *P* = 0.003). The absence of G6 was associated with a significant decrease in the MD° discrepancies (β = −5.30, *P* = 0.046) and MD-mm discrepancies (β = −3.21, *P* = 0.024). Crowding, OJ, gender, and the presence of different attachments (O1, H3, H4) did not demonstrate significant associations with any of the movement parameters (*P* > 0.05 for all). These findings highlight age, OB, TADs status, and G6 as key determinants for optimizing orthodontic treatment outcomes for the upper first molar.

### Upper second molar (U7)

The analysis for the upper second molar (Supplementary Table 9) revealed several significant associations. OB was significantly associated with an increase in the mesiodistal linear (MD-mm) movement discrepancies (β = 0.95, *P* < 0.001). Crowding showed a significant negative association with intrusion-extrusion linear (IE-mm) movement discrepancies (β = −0.08, *P* = 0.023). The absence of TADs was significantly associated with a decrease in the buccolingual angular (BL⁰) discrepancies (β = −7.12, *P* = 0.033). The presence of the 3 mm horizontal attachments (H3) was significantly associated with a decrease in the mesiodistal angular (MD⁰) discrepancies compared to the 3 mm vertical attachment (V3) (β = −11.46, *P* = 0.039). Age, OJ, gender, one optimized attachment (O1), and the absence of G6 did not demonstrate significant associations with any of the movement parameters (*P* > 0.05 for all). These findings underscore OB, crowding, TADs status, and attachment type as crucial factors influencing upper second molar movement, emphasizing their importance in achieving optimal orthodontic treatment outcomes.

**Table 1:** Baseline patient characteristics

Characteristics	Mean + SD/NO
Age (year)	24.73 ± 6.07
Gender:	
Male	11 (26.38 ± 6.48 years)
Female	22 (23.77 ± 5.76 years)
Overbite (mm)	2.27 ± 1.57
Overjet (mm)	4.42 ± 2.24
Crowding (mm):	
Upper arch	3.21 ± 3.34
Lower arch	4.13 ± 3.37

**Table 2:** Intra-class correlation coefficient of tooth movement measurement

Measurement	ICC*	95% CI^#^
**Angular measurements**:		
Mesiodistal angulation	0.9576	0.8511, 0.9901
Buccolingual inclination	0.9758	0.9242, 0.9925
**Linear measurements**:		
Mesiodistal movement	0.9658	0.8642, 0.9901
Buccolingual movement	0.9617	0.8771, 0.9832
Intrusion–Extrusion movement	0.9468	0.8352, 0.97581

*ICC interclass correlation coefficient. ^#^*CI* confidence intervals.

**Table 3:** Differences between designed and achieved linear and angular upper teeth movements

Tooth	Tooth movements	Mean ± SD	Mean Dif. ±SD	*P*-Value*
U1	MD⁰ designed	−3.75 ± 2.09	0.06 ± 2.57	0.873
	MD⁰ achieved	−3.81 ± 2.32		
	BL⁰ designed	39.34 ± 4.54	10.88 ± 4.55	0.000**
	BL⁰ achieved	28.46 ± 5.59		
	MD^mm^ designed	−9.07 ± 24.35	1.49 ± 3.29	0.002**
	MD^mm^ achieved	−10.56 ± 27.35		
	BL^mm^ designed	−0.01 ± 4.76	0.11 ± 0.71	0.208
	BL^mm^ achieved	−0.12 ± 4.79		
	IE^mm^ designed	0.36 ± 1.07	−0.42 ± 1.37	0.027*
	IE^mm^ achieved	0.77 ± 1.50		
U2	MD⁰ designed	−13.28 ± 4.21	1.71 ± 4.98	0.013*
	MD⁰ achieved	−14.99 ± 5.17		
	BL⁰ designed	36.32 ± 5.66	10.56 ± 5.57	0.000***
	BL⁰ achieved	25.76 ± 5.66		
	MD^mm^ designed	−8.33 ± 21.14	1.33 ± 3.01	0.002**
	MD^mm^ achieved	−9.66 ± 23.77		
	BL^mm^ designed	−0.18 ± 13.50	0.09 ± 0.93	0.622
	BL^mm^ achieved	−0.27 ± 13.54		
	IE^mm^ designed	0.34 ± 1.13	−0.01 ± 0.99	0.76
	IE^mm^ achieved	0.35 ± 1.22		
U3	MD⁰ designed	−5.87 ± 3.90	2.36 ± 4.21	0.000***
	MD⁰ achieved	−8.23 ± 4.34		
	BL⁰ designed	32.20 ± 4.88	10.92 ± 6.07	0.000***
	BL⁰ achieved	21.29 ± 4.93		
	MD^mm^ designed	−6.77 ± 16.81	0.51 ± 3.40	**0.053**
	MD^mm^ achieved	−7.28 ± 18.85		
	BL^mm^ designed	−0.08 ± 19.15	0.63 ± 4.92	0.941
	BL^mm^ achieved	−0.71 ± 18.95		
	IE^mm^ designed	0.51 ± 1.34	0.13 ± 1.07	0.358
	IE^mm^ achieved	0.38 ± 1.22		
U5	MD⁰ designed	3.69 ± 5.47	−8.44 ± 6.38	0.000***
	MD⁰ achieved	12.14 ± 5.53		
	BL⁰ designed	11.44 ± 4.29	8.75 ± 11.20	0.000***
	BL⁰ achieved	2.70 ± 10.74		
	MD^mm^ designed	−3.22 ± 9.39	1.03 ± 2.81	0.012*
	MD^mm^ achieved	−4.25 ± 11.77		
	BL^mm^ designed	−1.00 ± 24.06	−0.10 ± 0.90	0.211
	BL^mm^ achieved	−0.90 ± 23.77		
	IE^mm^ designed	0.69 ± 1.84	0.62 ± 1.46	0.002**
	IE^mm^ achieved	0.07 ± 0.96		
U6	MD⁰ designed	1.24 ± 3.69	−8.91 ± 5.04	0.000***
	MD⁰ achieved	10.15 ± 5.13		
	BL⁰ designed	4.07 ± 4.47	1.05 ± 4.93	0.116
	BL⁰ achieved	3.01 ± 6.13		
	MD^mm^ designed	−1.32 ± 3.47	0.67 ± 3.03	0.041*
	MD^mm^ achieved	−1.99 ± 5.69		
	BL^mm^ designed	−1.38 ± 26.71	−0.44 ± 2.75	0.223
	BL^mm^ achieved	−0.94 ± 26.96		
	IE^mm^ designed	0.44 ± 1.40	0.54 ± 1.39	0.005**
	IE^mm^ achieved	−0.10 ± 0.81		
U7	MD⁰ designed	−1.78 ± 6.35	−7.08 ± 6.60	0.000***
	MD⁰ achieved	5.30 ± 7.89		
	BL⁰ designed	9.12 ± 5.89	−2.13 ± 6.14	0.012*
	BL⁰ achieved	11.24 ± 9.24		
	MD^mm^ designed	2.71 ± 9.27	1.14 ± 3.44	0.016*
	MD^mm^ achieved	1.57 ± 7.40		
	BL^mm^ designed	−1.02 ± 29.61	0.10 ± 1.32	0.593
	BL^mm^ achieved	−1.11 ± 30.29		
	IE^mm^ designed	−0.18 ± 0.92	−0.08 ± 0.80	0.260
	IE^mm^ achieved	−0.10 ± 0.72		

Paired t-test P* (< 0.05), P** (< 0.01), P*** (< 0.001). Mesial tooth tipping is defined as positive (º), while distal tooth tipping is negative (º). Buccal tooth inclination is defined as positive (º), and lingual tooth inclination is defined as negative (º). Tooth extrusion defined as positive, and intrusion as negative (mm)

## Discussion

This study aimed to compare designed and achieved 3D whole tooth (crown with root) movements and their influencing factors for all maxillary teeth in first premolar extraction patients treated with Invisalign^®^. The CRAM model enhances orthodontic planning by combining crown, root, and alveolar bone data from intraoral and CBCT scans. Unlike traditional crown-only methods in ClinCheck^®^ software [5, 8, 10, 11], CRAM offers a more accurate, full-tooth view, improving prediction and minimizing side effects. It shows promise in complex cases like premolar extraction but needs further clinical validation.

Regarding using CBCT ethics, several studies support the use of CBCT in orthodontics when justified by clinical necessity, particularly when conventional imaging is insufficient. The justification for its use must adhere to strict criteria under the ALARA (As Low As Reasonably Achievable) principle, particularly in paediatric patients [12, 13]. At the same time, recent technological advances have introduced ultra-low-dose CBCT protocols that significantly reduce the radiation burden while maintaining diagnostic value [14]. In this context, several experts propose evolving from the ALARA principle to the ALADA (As Low As Diagnostically Acceptable) concept, which allows for better clinical outcomes without compromising patient safety [15].

### Upper teeth angular movements

The angular discrepancies between designed and achieved movements in the upper teeth revealed consistent deviations, particularly in mesiodistal tipping (MD⁰) and buccolingual inclination (BL⁰). Statistically significant differences in MD⁰ were noted for U2, U3, U5, U6, and U7 (*P* < 0.05 to *P* < 0.001), with anterior teeth (U2–U3) showing greater distal tipping and posterior teeth (U5–U7) more mesial tipping. This pattern suggests a “roller coaster effect” and highlights limitations in mesiodistal control using CA, necessitating the use of overcorrection strategies. These findings are consistent with studies reporting mesiodistal angulation TM inaccuracies with CA, often influenced by factors such as extraction space and root morphology [5–9]. Notably, U1 showed no significant difference in mesiodistal tipping, possibly due to simpler root morphology, attachment designs for central incisors [16], and their position near the center of the arch, which may reduce their susceptibility to the ‘roller coaster effect’ effect and associated distal tipping during retraction [17].

Regarding BL⁰, most upper teeth showed significant discrepancies (*P* < 0.05), except for U6. Most teeth demonstrated underachieved inclination along the whole tooth’s long axis, with less buccal crown tipping/lingual root torque than predicted. Previous studies reported that the BL⁰ overachieved inclination with more lingual crown tipping than predicted [5, 6, 8]. However, this inconsistency may be attributed to improved torque movement (TM) predictability with contemporary clear aligner systems (new tooth model, CRAM), as well as our assessment based on the whole tooth’s long axis. Moreover, the overcorrection strategies, power ridges, and attachment designs may improve torque control [8, 18, 19]. At the same time, U7 showed a slightly overexpressed inclination with more buccal crown tip/lingual root torque than predicted. This overexpression possibly reflects anatomical constraints (e.g., thicker alveolar bone) or differential force application in posterior regions [20, 21].

### Upper teeth linear movements

Significant MD-mm discrepancies were observed in U1, U2, U5, U6, and U7 (*P* < 0.05 to *P* < 0.01), with U1–U2 drifting more distally and U5–U7 showing more mesial shift. These overachievements mirror the findings of previous studies, where unanticipated crown drift in maxillary incisors and molars during extraction treatment with Invisalign^®^, likely due to aligner slack, suboptimal staging, or attachment design [6, 8]. The lack of significance in U3 implies better control for canines, potentially due to strategic attachment placement or applied overcorrection [7].

For BL-mm, no significant differences were found in any of the upper teeth (*P* > 0.05), indicating that the achieved buccolingual linear movements were consistent with the designed values. This may reflect the improvement of BL-mm in Invisalign^®^, as previous studies reported discrepancies in BL-mm [5, 6, 8].

However, vertical movement (IE-mm) showed significant deviations in U1, U5, and U6(*P* < 0.05 to *P* < 0.01). U1 was over-extruded while U5 and U6 underachieved their planned intrusion/extrusion, consistent with previous studies, which noted vertical control is challenged [5, 6, 8].

### Upper teeth movement influencing factors

The discrepancies between designed and achieved upper teeth movements in CA therapy are influenced by biomechanical, anatomical, and patient-specific factors. Below, we discuss these factors for each upper tooth type, supported by clinical evidence.

### Upper central incisor (U1)

For U1, increased OJ has been linked to greater mesiodistal deviation and tipping, likely due to amplified retraction forces acting on the incisor segment. This contrasts with the findings of Ren et al. who reported that greater OJ correlated with increased mesiodistal deviation in U3 but not in U1 among extraction cases [8]. Deep overbite has also been shown to impair torque control and buccolingual root positioning. Jeon et al. found that deep overbite was associated with decreased predictability of overbite correction and contributed to U1 overexpression TM in aligner cases [22]. Torque limitations were further exacerbated when using power ridges instead of optimized attachments, as confirmed by Rossini et al., who highlighted the mechanical limits of aligner-generated torque without precise auxiliary design [23]. Rajan et al. also found no significant differences in torque accuracy based on the presence of power ridges or aligner change protocols [24]. Gender differences were noted, with males exhibiting larger buccolingual deviation, potentially due to greater bone density, an effect also observed by Ren et al. in a multivariate analysis [8]. G6 showed minimized buccolingual errors, which may be related to its advancement in biomechanics ratherthan attachments design. For instance, Dai et al. found that G6 attachments do not outperform conventional horizontal attachments [5]. Our previous study also showed that no significant difference between G6 attachments and other conventional vertical rectangular attachments in achieving more predictable incisor tooth movements [8].

### Upper lateral incisor (U2)

For U2, deeper OB limits the capacity to control mesiodistal tipping while exacerbating buccolingual torque challenges, likely due to reduced vertical clearance and increased occlusal constraints. These findings are consistent with Blundell and Liu et al., who noted torque delivery limitations under vertical occlusal pressure [25, 26]. Additionally, the absence of optimized attachments or reliance on non-specific geometries like power ridges showed increased mesiodistal linear errors. Castroflorio et al. confirmed that optimized attachments improved angular and linear control in U2 [27]. Similar to findings in U1, male gender and G6 absence influenced buccolingual accuracy, reinforcing the role of anatomical and technological factors in lateral incisor control [5, 8].

### Upper canine (U3)

Older age was associated with reduced vertical movement predictability in U3, likely due to diminished bone remodelling capacity in older adults, a trend confirmed by Ren et al., who identified age as a predictor of TM in extraction-based clear aligner therapy [8]. The absence of TADs was associated with increased vertical (IE-mm) error, supporting findings by Dai et al. and Geramy et al., who showed that miniscrew anchorage improves vertical movement control [6, 28]. Additionally, optimized attachments (O1) performed worse than vertical rectangular designs (V3) in IE-mm accuracy, likely due to less effective force direction, consistent with the torque control insights of Feng et al. [7].

### Upper second premolar (U5)

Mesiodistal angular discrepancies in U5 were significantly influenced by dental crowding, likely due to interproximal resistance during space coordination, a trend confirmed by Ren et al. [8]. Additionally, reduced overjet was associated with increased mesiodistal linear discrepancies in U5, likely due to diminished anterior anchorage and force dissipation during space closure. Dai et al. observed that limited horizontal overlap can compromise anterior anchorage integrity, resulting in unintended mesial movement of posterior teeth during aligner-guided retraction mechanics [5]. Regarding attachment design, horizontal attachments (H3) resulted in slightly greater buccolingual discrepancies than vertical rectangular attachments (V3), suggesting that while H3 may assist in angulation, they may compromise torque precision. This observation parallels the network meta-analysis by Alam et al., which found that auxiliary attachment geometry significantly affects root torque and mesiodistal accuracy [29].

### Upper first molar (U6)

Vertical discrepancies (IE-mm) in U6 increased with patients’ age, likely due to slower bone remodeling, consistent with findings from Dai et al. and Ren et al. [5, 8]. Increased overbite was associated with larger mesiodistal discrepancies in U6, likely due to anterior occlusal interference that disrupts posterior distalization mechanics and contributes to anchorage loss. This pattern is consistent with findings from Ren et al., who identified overbite as a significant predictor of molar mesial drift, and Sadek et al., who reported occlusal interference as a contributing factor to unplanned tooth movement during deep bite correction [8, 30]. Generation 6 (G6) showed minimized mesiodistal discrepancies, which may reflect advancements in this protocol (G6 Reinforcement 2020, G6R). For instance, Dai et al. found that G6 attachments do not outperform conventional horizontal attachments in first molar angulation, and mesiodistal translation [5]. Our previous study also showed no difference between G6 attachments and conventional vertical rectangular attachments in achieving more predictable first molar angulation [8].

### Upper second molar (U7)

Crowding was associated with reduced vertical control (IE-mm), likely due to space constraints and interproximal resistance that impair force delivery and bodily movement. Ren et al. and Miao et al. reported that arch crowding significantly compromises distalization accuracy, especially in posterior teeth [8, 31]. Regarding attachment design, horizontal rectangular attachments (H3) provided better mesiodistal angular control than vertical attachments (V3), likely due to more effective engagement near the center of resistance. Thilagalavanian et al. recommended H3 for managing molar inclination during distalization, and Gao et al. confirmed through finite element modelling that horizontal attachments improve torque expression and angulation control in maxillary molars [9, 32].

Clinically, this study offers valuable guidance for managing complex maxillary tooth movements with Invisalign^®^ following first premolar extraction. Notably, significant deviations in mesiodistal tipping and buccolingual inclination highlight persistent challenges in root control during space closure. Overcorrection may be necessary, particularly for distal tipping in U2/U3 and mesial tipping in U5–U7, along with expected under expression of buccolingual torque in posterior teeth. The use of TADs improved mesiodistal linear predictability, supporting their strategic use to enhance anchorage. Treatment outcomes were also influenced by aligner features, such as Generation 6 (G6) protocols and auxiliary attachments, reinforcing the importance of tailored appliance selection. Patient-specific factors like deep overbite and gender were linked to reduced predictability in torque and linear control. These insights support a more individualized treatment planning approach, using root-based digital modelling to improve outcomes, especially in anterior torque and vertical control.

This study has several limitations. First, the sample size (33 patients, 396 teeth) and single-center retrospective design may limit generalizability, necessitating future multi-center studies with larger, diverse cohorts. Second, the crown rotation was not assessed because we focused on the roots rather than the crown movements. Third, our findings are specific to the Invisalign^®^ appliance and may not be generalizable to systems with different aligner materials, trimlines, or movement protocols. Fourth, biological variables such as bone density, patient compliance, and metabolic responses to aligner forces were not analysed, yet these factors likely influence movement accuracy. Fifth, the study focused solely on outcomes after the initial CA series; long-term stability and cumulative effects across multiple series remain unaddressed. Sixth, the findings are specific to first premolar extraction protocols and may not apply to non-extraction or other extraction patterns. Finally, the study cohort consisted exclusively of adults (mean age: 18–35 years), limiting applicability to adolescent populations with higher bone remodelling rates. Addressing these limitations in future research will strengthen clinical applicability.

## Conclusion

This study highlights significant deviations between designed and achieved maxillary teeth (crowns with roots) movements following first premolar extractions in the first series of Invisalign^®^ treatments. The new root digital model exposed root-level discrepancies undetectable by traditional crown-based analyses, underscoring the necessity of 3D root tracking in clinical planning. Key findings demonstrate significant mesiodistal (except U1) and buccolingual (all teeth) angular movement deviations. While linear buccolingual movements were well-controlled, mesiodistal and vertical (U1, U5, U6) discrepancies were more pronounced, influenced by factors such as TAD usage, aligner generation (G6), attachment design, overbite, and gender.

However, clinicians must adopt a proactive approach to treatment planning, incorporating patient-specific biomechanical strategies (e.g., TADs for anchorage, optimized attachments for torque) and anticipating overcorrection in high-risk movements. These findings advocate for personalized aligner design and highlight the need for further innovation in digital orthodontic workflows to bridge the gap between virtual planning and clinical execution. Future research should validate this model in diverse clinical scenarios and explore adaptive algorithms to improve predictability in complex extraction cases.

## Electronic supplementary material

Supplementary Material 1

## Data Availability

No datasets were generated or analysed during the current study.

## Abbreviations

CA: Clear aligner
FA: Fixed appliance
CRAM: Crown, root, and alveolar bone model
TADs: Temporary anchorage devices
G6: Sixth Invisalign^®^ generation
OJ: Overjet
OB: Overbite
H3: 3 mm horizontal attachment
H4: 4 mm horizontal attachment
V3: 3 mm vertical attachment
V4: 4 mm vertical attachment
O1: One optimized attachment
O2: Two optimized attachment
PR: Power ridge
MD⁰: Mesiodistal angulation
BL⁰: Buccolingual inclination
MD ^mm^: Mesiodistal translation
BL ^mm^: Buccolingual translation
IE ^mm^: Intrusion-extrusion translation
ICC: Intraclass correlation coefficient
TM: Tooth movement
