# Supplementary material for: 3D comparative analysis of designed versus achieved maxillary teeth movements and influencing factors following first premolar extraction with invisalign: a new digital root model
Source: Prog Orthod. 2025 Jul 1;26:21. doi: 10.1186/s40510-025-00569-z (PMC12214191; doi:10.1186/s40510-025-00569-z)
Supplement: Supplementary file 1 — Supplementary Material 1 [file 40510_2025_569_MOESM1_ESM.docx]

Supplementary Table 4  Multivariate linear mixed model regression analysis of the differences between designed and achieved tooth movements for upper central incisors.

| **Parameter** | **MD ⁰** | | **BL ⁰** | | **MD ^mm^** | | **BL ^mm^** | | **IE ^mm^** | |
| --- | --- | --- | --- | --- | --- | --- | --- | --- | --- | --- |
|  | **β**  **Estimate**  **(95% CI)** | **P** | **β**  **Estimate**  **(95% CI)** | **P** | **β**  **Estimate**  **(95% CI)** | **P** | **β**  **Estimate**  **(95% CI)** | **P** | **β**  **Estimate**  **(95% CI)** | **P** |
| **Age** | -0.01  (-0.11, 0.10) | 0.875 | 0.11  (-0.06, 0.28) | 0.203 | 0.00  (-0.07, 0.06) | 0.902 | 0.00  (-0.03, 0.03) | 0.933 | -0.01  (-0.08, 0.06) | 0.868 |
| **Overjet** | 0.34  (0.07, 0.62) | **0.015** | -0.39  (-0.84, 0.06) | 0.088 | 0.16  (-0.01, 0.34) | 0.070 | 0.06  (-0.02, 0.14) | 0.114 | -0.13  (-0.31, 0.04) | 0.134 |
| **Overbite** | -0.45  (-0.86, -0.04) | **0.032** | 1.19  (0.52, 1.86) | 0.001 | 0.01  (-0.27, 0.28) | 0.955 | -0.06  (-0.18, 0.05) | 0.266 | 0.31  (0.05, 0.56) | **0.022** |
| **Crowding** | -0.20  (-0.46, 0.06) | 0.126 | -0.35  (-0.76, 0.06) | 0.091 | 0.16  (0.00, 0.32) | 0.055 | 0.08  (0.01, 0.15) | **0.025** | 0.00  (-0.16, 0.17) | 0.956 |
| **[TAD=No]** | 0.31  (-1.62, 2.23) | 0.749 | 2.77  (-0.35, 5.89) | 0.081 | -1.79  (-3.01, -0.57) | **0.005** | 0.53  (0.01, 1.06) | **0.047** | 0.14  (-1.10, 1.39) | 0.820 |
| **[TAD=Yes]** | Reference | | | | | | | | | |
| **[Gender=Male]** | 0.17  (-1.27, 1.60) | 0.817 | 0.55  (-1.77, 2.86) | 0.638 | 0.36  (-0.57, 1.28) | 0.442 | 0.48  (0.09, 0.87) | **0.016** | -0.29  (-1.19, 0.61) | 0.518 |
| **[Gender=Female]** | Reference | | | | | | | | | |
| **[G6=No]** | 1.23  (-1.16, 3.63) | 0.305 | -5.63  (-9.55, -1.70) | **0.006** | -0.33  (-2.05, 1.39) | 0.700 | -1.34  (-2.00, -0.68) | **0.000** | -0.82  (-2.37, 0.72) | 0.289 |
| **[G6=Yes]** | Reference | | | | | | | | | |
| **[Attachment=No]** | 0.19  (-1.14, 1.52) | 0.777 | -3.41  (-5.58, -1.24) | **0.003** | -0.56  (-1.41, 0.29) | 0.189 | -0.41  (-0.77, -0.04) | **0.030** | -0.06  (-0.91, 0.80) | 0.896 |
| **[Power Ridge (PR)]** | Reference | | | | | | | | | |

Note. Bold values indicate statistical significance (*P* < 0.05).

Supplementary Table 5  Multivariate linear mixed model regression analysis of the differences between designed and achieved tooth movements for upper lateral incisors.

| **Parameter** | **MD ⁰** | | **BL ⁰** | | **MD ^mm^** | | **BL ^mm^** | | **IE ^mm^** | |
| --- | --- | --- | --- | --- | --- | --- | --- | --- | --- | --- |
|  | **β**  **Estimate**  **(95% CI)** | **P** | **β**  **Estimate**  **(95% CI)** | **P** | **β**  **Estimate**  **(95% CI)** | **P** | **β**  **Estimate**  **(95% CI)** | **P** | **β**  **Estimate**  **(95% CI)** | **P** |
| **Age** | 0.06  (-0.17, 0.29) | 0.603 | 0.09  (-0.18, 0.35) | 0.508 | -0.01  (-0.08, 0.05) | 0.672 | 0.01  (-0.03, 0.05) | 0.613 | 0.04  (-0.01, 0.08) | 0.131 |
| **Overjet** | 0.31  (-0.27, 0.88) | 0.288 | -0.46  (-1.11, 0.18) | 0.155 | 0.03  (-0.14, 0.21) | 0.703 | 0.06  (-0.05, 0.16) | 0.282 | 0.02  (-0.09, 0.14) | 0.706 |
| **Overbite** | -1.17  (-2.06, -0.28) | **0.011** | 1.25  (0.37, 2.14) | **0.007** | 0.24  (-0.05, 0.52) | 0.106 | 0.07  (-0.09, 0.23) | 0.376 | 0.08  (-0.09, 0.26) | 0.351 |
| **Crowding** | -0.24  (-0.80, 0.33) | 0.403 | 0.01  (-0.57, 0.58) | 0.983 | 0.12  (-0.05, 0.29) | 0.158 | 0.09  (-0.02, 0.19) | 0.094 | -0.09  (-0.20, 0.03) | 0.137 |
| **[TAD=No]** | 3.51  (-0.72, 7.74) | 0.101 | 4.81  (0.51, 9.11) | **0.029** | -2.40  (-3.68, -1.12) | **0.000** | 0.30  (-0.46, 1.06) | 0.432 | 0.64  (-0.23, 1.51) | 0.144 |
| **[TAD=Yes]** | Reference |  |  |  |  |  |  |  |  |  |
| **[Gender=Male]** | 0.07  (-3.27, 3.41) | 0.967 | -1.53  (-4.78, 1.72) | 0.348 | 0.45  (-0.52, 1.42) | 0.355 | 0.73  (0.16, 1.29) | **0.014** | -0.35  (-0.99, 0.28) | 0.269 |
| **[Gender=Female]** | Reference |  |  |  |  |  |  |  |  |  |
| **[G6=No]** | 0.91  (-4.19, 6.01) | 0.721 | -4.01  (-9.08, 1.05) | 0.117 | 0.05  (-1.64, 1.73) | 0.957 | -1.52  (-2.43, -0.61) | **0.002** | -0.04  (-1.08, 1.00) | 0.938 |
| **[G6=Yes]** | Reference |  |  |  |  |  |  |  |  |  |
| **[Attachment=No]** | -0.19  (-3.63, 3.24) | 0.910 | 1.07  (-2.57, 4.72) | 0.557 | 1.04  (0.01, 2.08) | **0.049** | 0.45  (-0.16, 1.07) | 0.144 | -0.52  (-1.20, 0.17) | 0.135 |
| **[Power Ridge (PR)]** | -0.13  (-3.50, 3.23) | 0.937 | 0.29  (-3.12, 3.69) | 0.867 | 1.17  (0.16, 2.19) | **0.024** | 0.41  (-0.19, 1.01) | 0.179 | -0.16  (-0.83, 0.51) | 0.639 |
| **[One Optimized Attachment (O1)]** | Reference |  |  |  |  |  |  |  |  |  |

Note. Bold values indicate statistical significance (*P* < 0.05).

Supplementary Table 6  Multivariate linear mixed model regression analysis of the differences between designed and achieved tooth movements for upper canines.

| **Parameter** | **MD ⁰** | | **BL ⁰** | | **MD ^mm^** | | **BL ^mm^** | | **IE ^mm^** | |
| --- | --- | --- | --- | --- | --- | --- | --- | --- | --- | --- |
|  | **β**  **Estimate**  **(95% CI)** | **P** | **β**  **Estimate**  **(95% CI)** | **P** | **β**  **Estimate**  **(95% CI)** | **P** | **β**  **Estimate**  **(95% CI)** | **P** | **β**  **Estimate**  **(95% CI)** | **P** |
| **Age** | 0.00  (-0.18, 0.19) | 0.969 | 0.09  (-0.17, 0.34) | 0.502 | -0.10  (-0.25, 0.05) | 0.200 | 0.18  (-0.08, 0.44) | 0.171 | 0.04  (0.00, 0.08) | **0.033** |
| **Overjet** | 0.13  (-0.38, 0.64) | 0.606 | -0.09  (-0.79, 0.62) | 0.809 | 0.12  (-0.31, 0.54) | 0.582 | -0.07  (-0.81, 0.66) | 0.841 | 0.09  (-0.01, 0.20) | 0.087 |
| **Overbite** | -1.08  (-1.77, -0.40) | **0.003** | 0.48  (-0.47, 1.44) | 0.312 | 0.32  (-0.30, 0.95) | 0.299 | -0.32  (-1.31, 0.68) | 0.528 | -0.09  (-0.24, 0.06) | 0.221 |
| **Crowding** | -0.09  (-0.60, 0.42) | 0.729 | -0.34  (-1.03, 0.34) | 0.318 | 0.10  (-0.31, 0.52) | 0.615 | -0.13  (-0.84, 0.59) | 0.726 | -0.09  (-0.20, 0.01) | 0.073 |
| **[TAD=No]** | -1.47  (-4.74, 1.80) | 0.371 | 1.14  (-3.87, 6.15) | 0.649 | -1.28  (-4.05, 1.49) | 0.356 | 1.14  (-3.66, 5.93) | 0.635 | 1.14  (0.42, 1.86) | **0.003** |
| **[TAD=Yes]** | Reference |  |  |  |  |  |  |  |  |  |
| **[Gender=Male]** | 0.34  (-1.97, 2.66) | 0.767 | -2.58  (-5.86, 0.71) | 0.121 | 0.27  (-1.73, 2.27) | 0.784 | -0.72  (-4.12, 2.68) | 0.671 | -0.73  (-1.23, -0.23) | **0.005** |
| **[Gender=Female]** | Reference |  |  |  |  |  |  |  |  |  |
| **[G6=No]** | 4.70  (0.74, 8.66) | **0.021** | 2.39  (-3.17, 7.95) | 0.391 | 0.02  (-3.65, 3.69) | 0.991 | -1.61  (-7.41, 4.19) | 0.578 | -0.30  (-1.15, 0.54) | 0.474 |
| **[G6=Yes]** | Reference |  |  |  |  |  |  |  |  |  |
| **[One Optimized Attachment (O1)]** | -1.73  (-5.64, 2.18) | 0.377 | 0.79  (-4.59, 6.16) | 0.770 | -0.29  (-3.48, 2.89) | 0.853 | 0.16  (-5.37, 5.69) | 0.953 | 1.27  (0.47, 2.08) | **0.003** |
| **[Two Optimized Attachment (O2)]** | 0.31  (-3.75, 4.37) | 0.879 | 2.42  (-3.43, 8.27) | 0.409 | 2.32  (-1.11, 5.74) | 0.180 | -0.70  (-6.63, 5.23) | 0.813 | 0.52  (-0.34, 1.38) | 0.227 |
| **[Vertical Attachment 3mm(V3)]** | Reference |  |  |  |  |  |  |  |  |  |

Note. Bold values indicate statistical significance (*P* < 0.05).

Supplementary Table 7  Multivariate linear mixed model regression analysis of the differences between designed and achieved tooth movements for upper second premolars.

| **Parameter** | **MD ⁰** | | **BL ⁰** | | **MD ^mm^** | | **BL ^mm^** | | **IE ^mm^** | |
| --- | --- | --- | --- | --- | --- | --- | --- | --- | --- | --- |
|  | **β**  **Estimate**  **(95% CI)** | **P** | **β**  **Estimate**  **(95% CI)** | **P** | **β**  **Estimate**  **(95% CI)** | **P** | **β**  **Estimate**  **(95% CI)** | **P** | **β**  **Estimate**  **(95% CI)** | **P** |
| **Age** | -0.18  (-0.45, 0.08) | 0.174 | 0.39  (-0.22, 0.99) | 0.208 | 0.07  (0.00, 0.15) | 0.065 | 0.03  (-0.02, 0.07) | 0.226 | -0.04  (-0.08, 0.00) | 0.075 |
| **Overjet** | 0.38  (-0.20, 0.96) | 0.193 | 0.71  (-0.67, 2.09) | 0.307 | -0.23  (-0.41, -0.06) | **0.011** | 0.08  (-0.02, 0.19) | 0.108 | -0.10  (-0.20, 0.00) | **0.048** |
| **Overbite** | 0.03  (-0.87, 0.93) | 0.944 | -0.36  (-2.63, 1.91) | 0.744 | 0.49  (0.19, 0.80) | **0.002** | -0.05  (-0.21, 0.11) | 0.512 | 0.10  (-0.05, 0.25) | 0.201 |
| **Crowding** | 1.08  (0.39, 1.77) | **0.003** | -0.15  (-1.77, 1.48) | 0.858 | -0.10  (-0.31, 0.11) | 0.349 | -0.01  (-0.13, 0.12) | 0.894 | 0.04  (-0.08, 0.16) | 0.493 |
| **[TAD=No]** | 3.63  (-0.69, 7.96) | 0.098 | -4.31  (-14.60, 5.99) | 0.404 | -1.94  (-3.27, -0.60) | **0.005** | -0.20  (-0.98, 0.59) | 0.613 | -0.45  (-1.17, 0.27) | 0.215 |
| **[TAD=Yes]** | Reference |  |  |  |  |  |  |  |  |  |
| **[Gender=Male]** | -2.79  (-5.83, 0.25) | 0.071 | -4.61  (-11.76, 2.55) | 0.202 | -0.15  (-1.10, 0.80) | 0.751 | 0.11  (-0.44, 0.66) | 0.680 | 0.18  (-0.32, 0.67) | 0.483 |
| **[Gender=Female]** | Reference |  |  |  |  |  |  |  |  |  |
| **[G6=No]** | -8.50  (-14.09, -2.90) | **0.004** | -2.67  (-15.76, 10.43) | 0.684 | -0.94  (-2.85, 0.96) | 0.323 | -0.20  (-1.21, 0.80) | 0.684 | 0.18  (-0.73, 1.09) | 0.696 |
| **[G6=Yes]** | Reference |  |  |  |  |  |  |  |  |  |
| **[Horizontal Attachment 3mm (H3)]** | -2.37  (-6.54, 1.79) | 0.257 | -9.66  (-19.68, 0.37) | 0.059 | 1.19  (-0.17, 2.55) | 0.086 | 0.77  (0.01, 1.53) | **0.047** | 0.63  (-0.05, 1.31) | 0.069 |
| **[One Optimized Attachment (O1)]** | -7.93  (-13.93, -1.94) | **0.011** | -2.50  (-16.62, 11.62) | 0.723 | 3.02  (1.18, 4.85) | **0.002** | 1.00  (-0.07, 2.07) | 0.067 | 0.44  (-0.56, 1.45) | 0.381 |
| **[Vertical Attachment 3mm**  **(V3)]** | Reference |  |  |  |  |  |  |  |  |  |

Note. Bold values indicate statistical significance (*P* < 0.05).

Supplementary Table 8  Multivariate linear mixed model regression analysis of the differences between designed and achieved tooth movements for upper first molars.

| **Parameter** | **MD ⁰** | | **BL ⁰** | | **MD ^mm^** | | **BL ^mm^** | | **IE ^mm^** | |
| --- | --- | --- | --- | --- | --- | --- | --- | --- | --- | --- |
|  | **β**  **Estimate**  **(95% CI)** | **P** | **Β**  **Estimate**  **(95% CI)** | **P** | **β**  **Estimate**  **(95% CI)** | **P** | **β**  **Estimate**  **(95% CI)** | **P** | **β**  **Estimate**  **(95% CI)** | **P** |
| **Age** | -0.12  (-0.37, 0.12) | 0.317 | 0.00  (-0.26, 0.27) | 0.972 | 0.09  (-0.04, 0.23) | 0.178 | 0.10  (-0.05, 0.26) | 0.199 | 0.05  (0.01, 0.09) | **0.020** |
| **Overjet** | 0.12  (-0.44, 0.68) | 0.658 | 0.06  (-0.56, 0.68) | 0.842 | -0.17  (-0.48, 0.14) | 0.279 | 0.23  (-0.13, 0.59) | 0.212 | 0.05  (-0.05, 0.15) | 0.319 |
| **Overbite** | 0.43  (-0.48, 1.33) | 0.346 | 0.31  (-0.68, 1.31) | 0.533 | 0.64  (0.12, 1.17) | **0.017** | -0.18  (-0.74, 0.37) | 0.516 | -0.09  (-0.25, 0.06) | 0.225 |
| **Crowding** | 0.58  (-0.07, 1.23) | 0.081 | -0.57  (-1.28, 0.14) | 0.114 | 0.06  (-0.30, 0.41) | 0.748 | -0.21  (-0.62, 0.21) | 0.326 | -0.08  (-0.19, 0.03) | 0.157 |
| **[TAD=No]** | 6.30  (2.23, 10.38) | **0.003** | -3.28  (-7.77, 1.22) | 0.149 | -1.73  (-3.95, 0.49) | 0.123 | -0.30  (-2.94, 2.35) | 0.823 | -0.12  (-0.85, 0.61) | 0.742 |
| **[TAD=Yes]** | Reference |  |  |  |  |  |  |  |  |  |
| **[Gender=Male]** | -1.92  (-4.87, 1.04) | 0.199 | 1.70  (-1.53, 4.93) | 0.294 | -0.80  (-2.41, 0.81) | 0.322 | -1.27  (-3.16, 0.63) | 0.185 | 0.01  (-0.51, 0.52) | 0.983 |
| **[Gender=Female]** | Reference |  |  |  |  |  |  |  |  |  |
| **[G6=No]** | -5.30  (-10.51, -0.10) | **0.046** | 2.80  (-2.71, 8.30) | 0.312 | -3.21  (-5.99, -0.43) | **0.024** | 1.54  (-1.68, 4.76) | 0.341 | 0.02  (-0.86, 0.89) | 0.969 |
| **[G6=Yes]** | Reference |  |  |  |  |  |  |  |  |  |
| **[[One Optimized Attachment (O1)]** | -3.73  (-10.08, 2.62) | 0.243 | 5.51  (-1.49, 12.51) | 0.120 | 2.74  (-0.72, 6.21) | 0.117 | 1.40  (-2.72, 5.52) | 0.498 | -0.10  (-1.22, 1.01) | 0.855 |
| **[Horizontal Attachment 3mm (H3)]** | -3.43  (-8.37, 1.51) | 0.169 | 4.40  (-1.00, 9.80) | 0.108 | 1.28  (-1.36, 3.91) | 0.334 | 0.22  (-2.91, 3.36) | 0.887 | -0.53  (-1.40, 0.33) | 0.221 |
| **[Horizontal Attachment 4mm (H4)]** | Reference |  |  |  |  |  |  |  |  |  |

Note. Bold values indicate statistical significance (*P* < 0.05).

Supplementary Table 9  Multivariate linear mixed model regression analysis of the differences between designed and achieved tooth movements for upper second molars.

| **Parameter** | **MD ⁰** | | **BL ⁰** | | **MD ^mm^** | | **BL ^mm^** | | **IE ^mm^** | |
| --- | --- | --- | --- | --- | --- | --- | --- | --- | --- | --- |
|  | **β**  **Estimate**  **(95% CI)** | **P** | **β Estimate**  **(95% CI)** | **P** | **β**  **Estimate**  **(95% CI)** | **P** | **β**  **Estimate**  **(95% CI)** | **P** | **β**  **Estimate**  **(95% CI)** | **P** |
| **Age** | -0.27  (-0.58, 0.04) | 0.085 | 0.20  (-0.11, 0.51) | 0.196 | -0.03  (-0.15, 0.09) | 0.625 | -0.01  (-0.06, 0.05) | 0.837 | 0.01  (-0.01, 0.04) | 0.348 |
| **Overjet** | 0.29  (-0.51, 1.09) | 0.467 | -0.06  (-0.85, 0.74) | 0.885 | -0.21  (-0.52, 0.10) | 0.173 | 0.09  (-0.06, 0.24) | 0.223 | -0.06  (-0.13, 0.01) | 0.081 |
| **Overbite** | -0.74  (-1.95, 0.47) | 0.225 | 0.59  (-0.57, 1.75) | 0.312 | 0.95  (0.49, 1.40) | **0.000** | 0.16  (-0.06, 0.38) | 0.147 | 0.02  (-0.08, 0.13) | 0.655 |
| **Crowding** | 0.70  (-0.12, 1.52) | 0.094 | -0.06  (-0.88, 0.76) | 0.885 | 0.03  (-0.29, 0.34) | 0.859 | -0.08  (-0.23, 0.08) | 0.323 | -0.08  (-0.16, -0.01) | **0.023** |
| **[TAD=No]** | 6.01  (-0.54, 12.56) | 0.071 | -7.12  (-13.66, -0.58) | **0.033** | -1.03  (-3.55, 1.50) | 0.416 | 0.05  (-1.19, 1.29) | 0.935 | -0.30  (-0.88, 0.29) | 0.309 |
| **[TAD=Yes]** | Reference |  |  |  |  |  |  |  |  |  |
| **[Gender=Male]** | -2.20  (-6.21, 1.80) | 0.274 | -0.04  (-3.97, 3.88) | 0.982 | -0.06  (-1.60, 1.48) | 0.937 | 0.45  (-0.30, 1.19) | 0.231 | 0.23  (-0.13, 0.59) | 0.196 |
| **[Gender=Female]** | Reference |  |  |  |  |  |  |  |  |  |
| **[G6=No]** | -3.98  (-10.77, 2.82) | 0.245 | -0.37  (-7.14, 6.41) | 0.914 | -1.01  (-3.92, 1.89) | 0.486 | -0.04  (-1.33, 1.24) | 0.947 | 0.09  (-0.50, 0.68) | 0.767 |
| **[G6=Yes]** | Reference |  |  |  |  |  |  |  |  |  |
| **[Horizontal Attachment 3mm (H3)]** | -11.46  (-22.30, -0.63) | **0.039** | 0.04  (-10.69, 10.77) | 0.994 | 1.63  (-2.50, 5.75) | 0.431 | 1.00  (-1.03, 3.04) | 0.326 | -0.11  (-1.06, 0.84) | 0.818 |
| **[One Optimized Attachment (O1)]** | -12.06  (-25.00, 0.88) | 0.067 | 0.75  (-11.98, 13.48) | 0.906 | 2.15  (-2.77, 7.06) | 0.384 | 2.00  (-0.42, 4.41) | 0.102 | 0.77  (-0.34, 1.88) | 0.170 |
| **[Vertical Attachment3mm**  **(V3)]** | Reference |  |  |  |  |  |  |  |  |  |

Note. Bold values indicate statistical significance (*P* < 0.05)
